# *NAC103* mutation alleviates DNA damage in an *Arabidopsis thaliana* mutant sensitive to excess boron

**DOI:** 10.3389/fpls.2023.1099816

**Published:** 2023-03-29

**Authors:** Naoyuki Sotta, Takuya Sakamoto, Takehiro Kamiya, Ryo Tabata, Katsushi Yamaguchi, Shuji Shigenobu, Masashi Yamada, Mitsuyasu Hasebe, Shinichiro Sawa, Toru Fujiwara

**Affiliations:** ^1^ Department of Applied Biological Chemistry, Graduate School of Agricultural and Life Sciences, The University of Tokyo, Tokyo, Japan; ^2^ Department of Applied Biological Science, Faculty of Science and Technology, Tokyo University of Science, Tokyo, Japan; ^3^ Graduate School of Science and Technology, Kumamoto University, Kumamoto, Japan; ^4^ Graduate School of Bioagricultural Sciences, Nagoya University, Nagoya, Japan; ^5^ National Institutes for Basic Biology, National Institutes of Natural Sciences, Okazaki, Japan; ^6^ School of Life Science, Graduate University for Advanced Studies, Okazaki, Japan; ^7^ Agricultural Biotechnology Research Center, Academia Sinica, Taipei, Taiwan; ^8^ Biotechnology Center in Southern Taiwan, Academia Sinica, Tainan, Taiwan; ^9^ International Research Center for Agricultural & Environmental Biology, Kumamoto University, Kumamoto, Japan

**Keywords:** abiotic stress, *Arabidopsis thaliana*, DNA repair, meristems, reactive oxygen species, root, stress response

## Abstract

Excess boron (B) is toxic to plants and thereby causes DNA damage and cell death in root meristems. However, the underlying mechanisms which link boron and DNA damage remain unclear. It has been reported that the *rpt5a-6* mutant of the 26S proteasome is sensitive to excess boron, resulting in more frequent cell death in root meristem and reduced root elongation. In this study, we showed that a reduction in root growth in the *rpt5a* mutant in the presence of high boron levels is repressed by a mutation in NAC domain containing transcription factor NAC103, a substrate of the proteasome, which functions in the unfolded protein response pathway. The mutation in NAC103 alleviated excess-B-induced DNA damage and cell death in root meristems of the *rpt5a* mutant. Superoxide ( $O_{2}^{-}$ ) staining with nitroblue tetrazolium revealed that boron stress causes $O_{2}^{-}$ accumulation in root tips, which was higher in the *rpt5a-6* mutant, whereas the accumulation was lower in the *rpt5a-6 nac103-3* double mutant. Our work demonstrates the overall involvement of *NAC103* in maintaining healthy root meristem under excess boron conditions in the absence of *RPT5A* proteasome subunit.

## 1 Introduction

Boron is an essential element for plants but is also toxic to them at high concentrations (Warington, 1923; Nable et al., 1997). Excess boron causes reduced root and shoot growth (Reid et al., 2004), and deformities in fruit growth (Dye et al., 1983; Sinha et al., 2006). Because excess boron can cause such defects in crops, the issue has become a significant agricultural problem. Thus, it is important to understand the mechanisms of plant tolerance to boron toxicity. In *Arabidopsis thaliana* roots, excess boron inhibits cell division and causes cell death, resulting in a reduced root meristem size and a slower root elongation (Aquea et al., 2012). Excess boron induces DNA damage within the root tip, and this phenomenon has been suggested to be a mechanism for the growth defects in the presence of excess boron (Sakamoto et al., 2011). The molecular mechanisms involved in DNA damage caused by boron stress are relatively unknown; however, recently we reported that the 26S proteasome is crucial for excess boron tolerance and that several 26S proteasome component genes are essential for ameliorating excess boron-induced DNA damage (Sakamoto et al., 2018).

26S proteasome is a protein complex which has a key function in selective proteolysis carried out by the ubiquitin (Ub)–proteasome pathway (Smalle and Vierstra, 2004). The Ub–proteasome pathway is a core mechanism for protein breakdown and is conserved in eukaryote species. In the pathway, target proteins were tagged with Ub, a highly conserved 76-amino acid protein (Smalle and Vierstra, 2004). The tagging is catalyzed by an ATP-dependent cascade involving the Ub-activating enzyme (E1), Ub-conjugating enzyme (E2), and Ub-protein ligase (E3) (Vierstra, 2003). The fact that *A. thaliana* genome encodes approximately 1200 E3 components suggests the involvement of the Ub-proteasome pathway in a wide range of biological processes and having different specificities. In fact, the Ub-proteasome pathway has been found to be important in a wide variety of physiological processes including cell division, differentiation, and development; stress response and extracellular modulation; morphogenesis of neuronal networks; cell surface receptor modulation; ion channeling and secretion pathways; DNA repair; regulation of the immune and inflammatory response; and, organelle biogenesis and apoptosis (Ciechanover, 1998).

The 26S proteasome consists of two subunits, the 20S proteasome [core particle (CP)] and the 19S complex [regulatory particle (RP)] (Kurepa and Smalle, 2008). Typically, the CP consists of two pairs of α and β subunits that form a barrel-like structure containing three sites for protease activity. The RP is located at the end of the CP barrel and forms a gate- cap structure through which the substrate proteins enter the CP. The RP consists of a base and lid. The base contains three RP non-ATPases (RPNs), namely RPN1, RPN2, and RPN10 and six RP AAA-ATPases (RPTs), i.e., RPT1–RPT6) (Fu et al., 2001). RPTs have an ATP-dependent function in the unfolding of substrate proteins and opening of the gate in order to allow the substrate to enter the CP (Wolf and Hilt, 2004).

RPT2A, RPT5A, RPN2A, and RPN8a have been identified as essential subunits playing a role in excess boron tolerance (Sakamoto et al., 2018). Mutants of the abovementioned subunits exhibit a significantly reduced root growth, stunned root meristems, increase in the number of root hairs, and frequent death of root meristematic cells under excess boron conditions. In the *rpt5a-4* mutant, a reduction in Ub–proteasome pathway activity and an overaccumulation of *BRAHMA* (*BRM*), a substrate of the Ub–proteasome pathway, were observed (Sakamoto et al., 2018). Because *BRM* is an SWI/SNF chromatin remodeling ATPase, sensitivity of the *rpt5a-4* mutant to excess boron was partially explained by a failure in BRM protein degradation, which resulted in over-loosening of chromatin and made the DNA more susceptible to damage. However, introducing the *brm* mutation only partially increased the tolerance of the *rpt5a-4* mutant to excess boron. This suggests that the *RPT5A* is required for excess boron tolerance and is also part of a pathway different to that used to degrade the BRM protein. To further the limited knowledge on molecular mechanisms underlying plant tolerance to excess boron, we screened for suppressor mutants of the *rpt5a* mutation in *A. thaliana*. In this work, we report characterization of several *rpt5a* suppressor lines and identification of a transcription factor which is involved in excess boron sensitivity in plant root growth.

## 2 Materials and methods

### 2.1 Plant materials and growth conditions

For screening *rpt5a* suppressor mutants, approximately 20,000 *rpt5a-4* and *rpt5a-6* seeds were treated with 0.2% ethyl methanesulfonate (EMS) for 16 h; then, those seeds were sown and grown on rockwool. The harvested seeds were cultured to produce M_2_ seeds that were subsequently harvested. Approximately 20,000 M_2_ seedlings were grown for 10 days in a 3 mM B MGRL media (Fujiwara et al., 1992) supplemented with 1.0% sucrose and solidified using 1.5% gellan gum (Wako, Japan). Plants that exhibited longer roots than those of the parental mutant were selected and transferred to 30 µM B plates for incubation. After 2–3 days of incubation, the selected plants were transferred to a rockwool, and the M_3_ seeds were eventually harvested. The phenotypes of the selected suppressor lines were confirmed with the seedlings of the M_3_ generation.

To obtain the *nac103-3* single mutant, the *rpt5a-6 nac103-3* mutant was backcrossed with the wild type to create a selfed-F_2_ population. The *nac103-3* mutation was detected by a CAPS marker after performing amplification of the genome using the primers nac103-3_F and nac103-3_R followed by digestion using *Hin*fI; the resultant band sizes were as follows: wild type: 234; *nac103-3*: 66, 168 bp. The *rpt5a-6* mutation was detected by a dCAPS marker after performing amplification using the primers rpt5a-6_BfuAI_F and rpt5a-6_R followed by digestion using *Bfu*IA; the resultant band sizes were as follows: wild type: 254, 297, 401; *rpt5a-6*: 254, 698 bp.

To obtain mutant combination of *rpt5a-6*, *nac103-3*, and *sog1-101*, *rpt5a-6 nac103-3* double mutant was crossed with *sog1-101* mutant, and homozygous lines for the genotype of interests were established from their F_2_ generation. *sog1-101* mutation was detected by PCR using the specific primers as described previously (Ogita et al., 2018).

For the growth tests, seeds were surface sterilized using 70% ethanol aq. For 5 min followed by 99.5% ethanol aq. For 1 min. After rinsing off the ethanol, the seeds were sown on a sterile MGRL medium supplemented with 1.0% sucrose and solidified using gellan gum (Wako). Boric acid concentrations in the medium were modified from the original concentration for each specific test. After vernalization at 4°C for 2 days, the medium-containing plates were placed vertically in incubators at 22°C under a 16-h light (fluorescent lamps, 100–160 μmol photons m^−2^ s^−1^) and 8-h dark cycle.

### 2.2 Positional identification of the causal mutation gene

The positional identification of the causal mutation gene was conducted by utilizing the polymorphism between the two ecotypes, Col-0 and L*er*. To obtain the F_2_-segregating generation, *rpt5a-6 sup1* (Col-0 background) mutant was crossed with L*er*. The F_1_ was self-pollinated, and the obtained F_2_ seeds were used for mapping of the gene responsible for the altered phenotype in root growth under excess boron condition. The F_2_ seedlings were grown under excess boron conditions (3 mM boric acid), and seedlings with short hairy roots similar to the *rpt5a-6* phenotype were selected for genotyping. The presence of *rpt5a-6* mutations in the mapping population was confirmed by the CAPS marker, as described above. For genotyping, a single-sequence length polymorphism (SSLP) marker between Col-0 and L*er* was used. A list of genetic markers near the candidate region used for mapping is shown in **Table S1**.

### 2.3 Plasmid construction and transformation of plants

For the genetic complementation tests, the *NAC103* genomic sequence without the termination codon was cloned from the Col-0 genomic DNA using the primers NAC103ATG-2k_F and NAC103CDS-TAA_R with a PrimeSTAR^®^ GXL DNA Polymerase (Takara). The amplified fragment contained an upstream region of 1,589 bp in size beginning at the annotated transcription start sites. The fragment was cloned into pENTR D-TOPO (Invitrogen) according to the instructions in the manufacturer’s manual. The obtained entry vector was designated as pSOT1. The entire insert sequences in the entry vector were verified by sequencing analysis. Sequences of primers are listed in **Table S1**. The vector for GFP fusion protein expression in plants was obtained by LR reaction between pSOT1 and pMDC107 (Curtis and Grossniklaus, 2003) using the gateway LR clonase enzyme mix (Invitrogen). The resultant expression vector carrying *proNAC103*-*NAC103*-*GFP* construct was designated as pSOT2. In a similar way, for NAC103 overexpression, the coding sequence of *NAC103* was cloned from the cDNA derived mRNA isolated from Col-0 roots into pENTR D-TOPO using the primers NAC103_CDS_F and NAC103_CDS_R and then transferred into the expression vector, pMDC32 (Curtis and Grossniklaus, 2003). The resultant expression vector carrying the *pro35S-NAC103* construct was designated as pSOT3.

For the stable transformation of the experimental plants, the binary vectors were introduced into the *Agrobacterium tumefaciens* strain GV3101(Bechtold and Pelletier, 1998), and the target plants were transformed with this culture using the floral dipping method (Clough and Bent, 1998). The transformed plants were selected on a half-strength MS medium containing 20 µg/mL hygromycin B (Wako) and 250 µg/mL Claforan (Sanofi), which was solidified using 0.5% agarose. Expression of the transgene was confirmed by qRT-PCR.

### 2.4 ROS staining

The superoxide was visualized by nitroblue tetrazolium (NBT) staining. Roots were incubated with an NBT staining solution (2 mM NBT in 20 mM MES, pH 6.1) for 5 min at room temperature in the dark. The roots were washed with water after staining and then mounted in chloral hydrate solution (a mixture of 4-g chloral hydrate, 1-mL glycerol, and 2-mL water). These roots were observed under a bright field microscope.

### 2.5 qRT-PCR analysis

For RNA extraction, 6-day-old wild-type seedlings were grown under control conditions (30 µM boric acid) and were either subsequently subjected to excess boron conditions (3 mM boric acid) or maintained under control conditions for an additional 3 days. The whole roots were harvested and subjected to RNA extraction using a RNeasy Plant Mini Kit (Qiagen). For qRT-PCR analysis, approximately 500 ng of total RNA was reverse transcribed using a Takara Prime Script RT Master Mix according to the manufacturer’s instructions at a 10-μL scale. Real-time PCR was performed using a Takara Thermal Cycler Dice TP800 and Takara SYBR^®^ Premix Ex Taq™ II (Tli RNaseH Plus) according to the manufacturer’s instructions. For amplification, the PCR cycle was set at 95°C for 30 s, 40 cycles of (95°C for 5s, 60°C for 30s^*^) for amplification, and then 95°C for 15s, 60°C for 30s, and 95°C for 15s^*^ for dissociation curve analysis. The fluorescence intensity was recorded at the steps marked with asterisks, and the primers used are shown in **Table S1**.

### 2.6 Confocal microscopy

For observation of the root meristems, approximately 5-mm regions from the root tips were excised and mounted in 10-µg/mL propidium iodide (PI) aq. For visualizing cell walls. The prepared slides were immediately observed under a confocal microscope (FV1000, OLYMPUS), which is equipped with a 40x objective lens, and subjected to excitation at 556 nm; the resulting signal intensity was detected between 570–670 nm.

### 2.7 Root growth measurement

Root images were captured using a digital camera, and root length was measured using the “Segmented Line” function in FIJI software (Schindelin et al., 2012).

### 2.8 DNA damage quantification

For the comet assay, nuclei were extracted from 100 primary root tips (1 cm from the tip) for every single replicate. Roots were chopped in 300 µL of nuclei extraction buffer (50 mM EDTA in PBS) using a razor. The buffer was collected and passed through a 30-µm membrane filter (CellTrics^®^, Sysmex Partec) to remove large cellular structures. Comet assay was performed using CometAssay^®^ Kit (25 x 2-well slides; Trevigen) by following the instructions in the kit’s manual and performing slight modifications: electrophoresis was performed at 1 V/cm for 6 min in an ice-cooled TBE buffer. For DNA staining, SYBR^®^ Gold Nucleic Acid Gel Stain (10,000x Concentrate in DMSO; Thermo Fisher) was used after diluting it by 30,000 times using TE buffer (pH 8.0). The prepared slides were observed under a fluorescent microscope (BX50WI, OLYMPUS), which was equipped with a U-MWIBA3 mirror unit (460–495 nm excitation, 510–550 nm absorbance, and a 505-nm dichroic mirror). Image quantification was performed by using default settings of CASP software (Końca et al., 2003).

### 2.9 Statistical analysis

For root length and elongation measurements, primary roots of 9-24 individuals were measured. For qRT-PCR, roots of 10 seedlings were sampled as one biological replicate, and three biological replicates were analyzed. For comet assay, nuclei were extracted from 100 seedlings as one sample, and 125 nuclei were measured for each sample. Dunnett’s test was applied for multiple comparison against control or wild-type. Tukey-Kramer’s test was applied for multiple comparison among all groups.

## 3 Results

### 3.1 Isolation of *rpt5a-6* suppressor mutants

**Figure 1:**
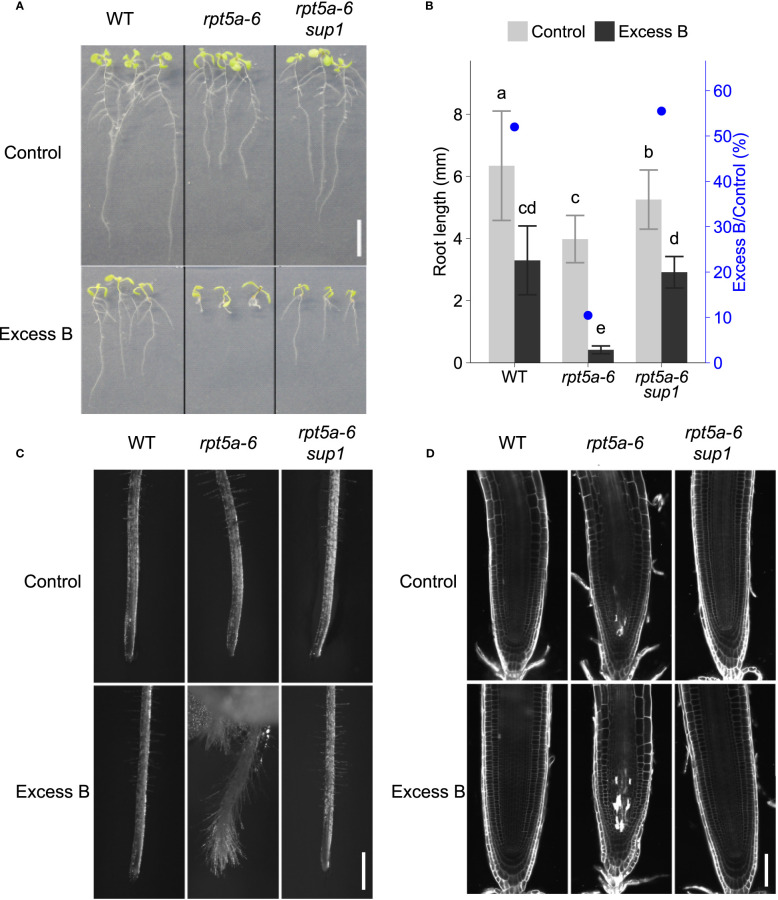
Phenotypes of the mutant in which excess boron sensitivity of *rpt5a-6* is suppressed. Wild type, *rpt5a-6* and its suppressor mutant *rpt5a-6 sup1* were grown under excess boron (3 mM boric acid) or control (30 µM boric acid) conditions. **(A)** Ten-day-old seedlings. Bar, 1cm. **(B)** Primary root length of ten-day-old seedlings. Values are mean ± standard deviation of 12-23 individuals. Blue dots represent relative root length. Groups sharing the same letters are not significantly different at *p* < 0.05 by Tukey-Kramer’s test. **(C)** Primary root tip morphology of 11-day-old seedlings by stereomicroscopy. Bar, 500 µm. **(D)** Confocal microscopy of root meristems. Six-day-old seedlings grown under the control condition were treated with control or excess boron conditions for 40 hours. Cell walls and dead cells were visualized with propidium iodide. Bar, 50 µm.

It has been reported that mutants of a subunit of the 26S proteasome, the *RPT5A* gene, are sensitive to excess boron (Sakamoto et al., 2018). Under excess boron conditions, mutants exhibit a severely inhibited root elongation phenotype, which is restored under normal boron conditions. To obtain insights into the role of the *RPT5A* gene with regard to excess boron tolerance, we screened for its suppressor mutants from M_2_ population of mutagenized *rpt5a-6*, a non-sense mutant, and *rpt5a-4*, a knock-out mutant. From the M_2_ *rpt5a-6* population, we isolated a suppressor line, *rpt5a-6 sup1*, the root elongation of which under conditions of boron stress (3 mM boric acid) was greater than that of the original mutant *rpt5a-6* (**Figure 1A**). Under normal conditions, roots of *rpt5a-6 sup1* was significantly longer than that of *rpt5a-6*, but shorter than the wild type, suggesting that *sup1* mutation suppresses not all the growth defects in *rpt5a-6* (**Figure 1B**). To evaluate excess boron sensitivity, apart from basic growth defects, we compared relative root growth (excess boron/control) among the genotypes. Relative root growth of *rpt5a-6 sup1* was higher than *rpt5a-6* and comparable to the wild type, suggesting that *sup1* mutation suppresses the excess boron sensitivity of *rpt5a-6* to the same levels as the wild type.

Previously, *rpt5a-6* had been found to exhibit an altered root morphology with short and squeezed root apical meristems and frequent cell death in the meristem even under normal boron conditions; these features become more evident by excess boron stress (Sakamoto et al., 2018). In *rpt5a-6 sup1*, the abnormal root meristem shape under excess boron stress was recovered and indistinguishable from the wild type (**Figure 1C**). Visualization of cell walls and dead cells using propidium iodide (PI) stain revealed that cell death is evident in stele cells in the meristem of *rpt5a* mutants after 40 h of excess boron treatment, and that the cell death was suppressed in *rpt5a-6 sup1* (**Figure 1D** and **Supplementary Figure 1**). From these results, we concluded that *rpt5a-6 sup1* completely suppresses the root growth sensitivity observed in *rpt5a-6* mutants to excess boron.

### 3.2 *NAC103* gene responsible for suppressing excess boron sensitivity in *rpt5a* mutants

**Figure 2:**
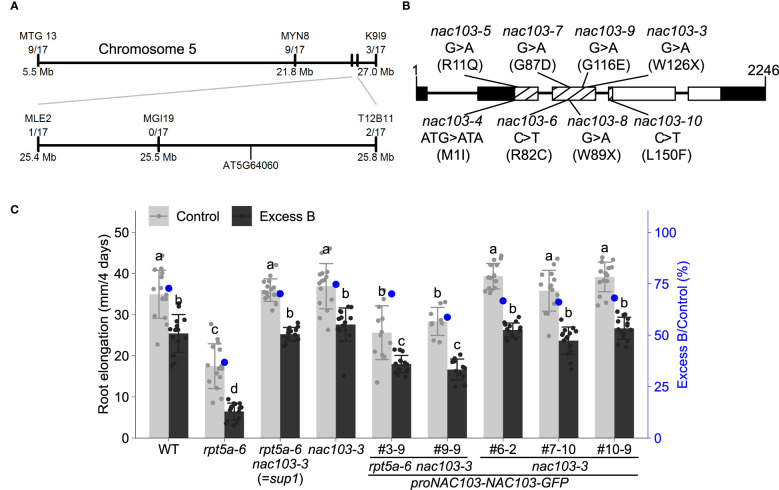
Positional identification of the mutation responsible for suppression of excess boron sensitivity in *rpt5a-6 sup1*. **(A)** Schematic diagram for candidate region in chromosome 5. Molecular markers and the number of recombinant plants found in the mapping population are shown. **(B)** Schematic diagram of *NAC103* gene and point mutations in suppressor lines. For each suppressor line, the line names, nucleotide changes and amino acid changes (parenthesis) are shown. Lines and boxes represent introns and exons respectively. White and black boxes represent cording regions and untranslated regions, respectively. NAC domain is shown by meshing. **(C)** Root elongation of the complementation lines. Constructs carrying *proNAC103-NAC103-GFP* was introduced into *rpt5a-6 nac103-3* or *nac103-3.* Seedlings were grown under normal conditions (30 µM boric acid) for eight days and then transferred to excess boron (3 mM boric acid) or control conditions. Root elongation during for four days after the transfer was measured. Numbering with # indicates transformant lines isolated independently. Values are mean ± standard deviation of 9-15 measurements. Groups sharing the same letters are not significantly different at *p* < 0.05 by Tukey’s test.

Crossing between the *rpt5a-6 sup1* and *rpt5a-4* mutants suggested that the *rpt5a-6 sup1* mutation is semi-dominant because the F1-progeny exhibited intermediate root growth between *rpt5a-6 sup1* and *rpt5a-4* (**Supplementary Figure 2**). Map-based cloning revealed that the causal gene for the suppression of excess boron sensitivity in *rpt5a-6 sup1* is located on chromosome 5, between 25.4 Mb and 25.8 Mb (**Figure 2A**). Within the chromosomal region, whole genome sequencing identified only a single homozygous mutation. The mutation was in the NAC transcription factor family, *NAC103* (AT5G64060), and is predicted to produce a premature stop codon in its coding region. We designated the mutation as *nac103-3* (**Figure 2B**). In addition, we isolated other 11 independent suppressor lines from the M_2_ *rpt5a-4* population (**Supplementary Figure 3**). These lines exhibited phenotypes similar to those exhibited by *rpt5a-6 nac103-3*. We determined the *NAC103* genomic sequence in the 11 *rpt5a-4* mutant suppressor lines and found that seven lines harbored a distinct mutation in the *NAC103* coding sequence, designated as *nac103-4, 5, 6, 7, 8, 9, and 10* (**Figures 2B**, **S2**). The presence of several alleles exhibiting similar phenotypes strongly suggests that *NAC103* is the gene responsible for the suppression. Because the phenotype was comparable among the abovementioned alleles, we focused on *rpt5a-6 nac103-3* for further analysis.

To further confirm that the mutation in *NAC103* is responsible for the suppression of the *rpt5a-6* phenotype, we conducted complementation tests. A construct carrying *proNAC103-NAC103-GFP* was introduced into *rpt5a-6 nac103-3*, and two independent homozygous transgenic lines were established. A qRT-PCR analysis confirmed that those lines accumulated higher or comparable *NAC103* mRNA compared to *rpt5a-6.* Under both normal boron concentration (control) and excess boron concentration, root elongation of these transformants was found to be significantly reduced in comparison with that of *rpt5a-6 nac103-3* (**Figure 2C**). To examine the effect of expressing the *NAC103* gene without *rpt5a-6* mutation, we also introduced the same construct into the *nac103-3* single mutant. The *nac103-3* single mutant was generated by backcrossing *rpt5a-6 nac103-3* with the wild type. The root elongation phenotype of the established *nac103-3* single mutant was indistinguishable from that of the wild type (**Figure 2C**). In contrast to the *rpt5a-6 nac103-3 proNAC103-NAC103-GFP* lines, the three transformant lines of *nac103-3 proNAC103-NAC103-GFP* exhibited root growth indistinguishable to that of *nac103-3*, thereby indicating that growth defects caused by the introduction of *NAC103* is specific to an *rpt5a-6* background. Given that the expression of *NAC103-GFP* in the *rpt5a-6 nac103-3* mutant resulted in the inhibition of root elongation in the *rpt5a-6* mutant, we concluded that *NAC103* is at least partly responsible for the phenotype of the *rpt5a-6* suppressor mutant.

### 3.3 Expression of *NAC103* gene induced by boron stress

**Figure 3:**
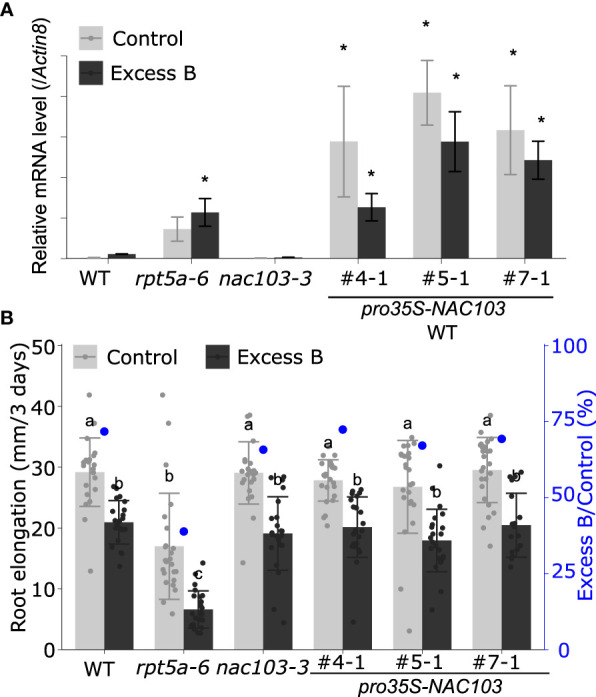
Effect of *NAC103* expression on excess boron sensitivity in root growth. **(A)** *NAC103* mRNA accumulation in *rpt5a-6* mutant and *NAC103* overexpression lines. Seedlings were grown for six days under control or excess boron conditions. Total RNA was extracted from whole root and *NAC103* mRNA accumulation was quantified by qRT-PCR. Expression levels were normalized by *Actin8*. Values represent mean ± standard deviation of three biological replicates. Asterisk indicate significant difference from the wild type in the same condition at *p* < 0.05 by Dunnett’s test. **(B)** Root elongation of *NAC103* overexpression lines. Seedlings were precultured in normal conditions (30 µM boric acid) for six days and then transferred to excess boron (3 mM boric acid) or control conditions. Root elongation during three days after the transfer was measured. Numbering with # indicates transformant lines isolated independently. Values are mean ± standard deviation of 18-24 measurements. Groups sharing the same letters are not significantly different at *p* < 0.05 by Tukey-Kramer’s test.

To investigate the reaction of the *NAC103* gene to boron stress, we examined the accumulation of *NAC103* mRNA in the *rpt5a-6* mutant in response to conditions of excess boron. RNA was extracted from whole roots of seedlings treated with excess boron (3 mM boric acid) and from those of seedlings grown under control conditions (30 µM boric acid) for 3 days. In the wild type, excess boron significantly induced the *NAC103* mRNA level by more than 10-fold (**Figure 3A**). mRNA accumulation was much higher in *rpt5a-6* plants grown under both conditions, having more than 50-fold increase in comparison with that in the wild-type plants grown under control condition. These results suggest the possibility that the overaccumulation of *NAC103* mRNA is responsible for root growth inhibition under conditions of excess boron in the *rpt5a-6* mutant.

### 3.4 *NAC103* overexpression in the wild type background did not induce root growth inhibition under excess boron condition

We hypothesized that an over accumulation of *NAC103* mRNA is responsible for root growth inhibition under excess boron stress. To test the hypothesis, we generated *NAC103* overexpression lines to observe their phenotypes under excess boron stress. For the *NAC103* overexpression lines, the coding sequence of *NAC103* was cloned from cDNA and introduced into the wild type under the control of the cauliflower mosaic virus 35S RNA promoter. Three independent transformant lines were established. A qRT-PCR analysis confirmed that those lines accumulated higher *NAC103* mRNA than the wild type as well as the *rpt5a-6* mutant, with a tendency to reduce under excess boron conditions for unknown reasons (**Figure 3A**). However, root growth in the overexpressing lines was indistinguishable from that in the wild type under both the excess boron and control conditions (**Figure 3B**). These results suggest that growth defects caused by excess boron are not explained by *NAC103* mRNA over accumulation alone.

### 3.5 No significant difference was observed between *rpt5a-6* and *rpt5a-6 nac103-3* mutants regarding the expression of already known NAC103-downstream genes

**Figure 4:**
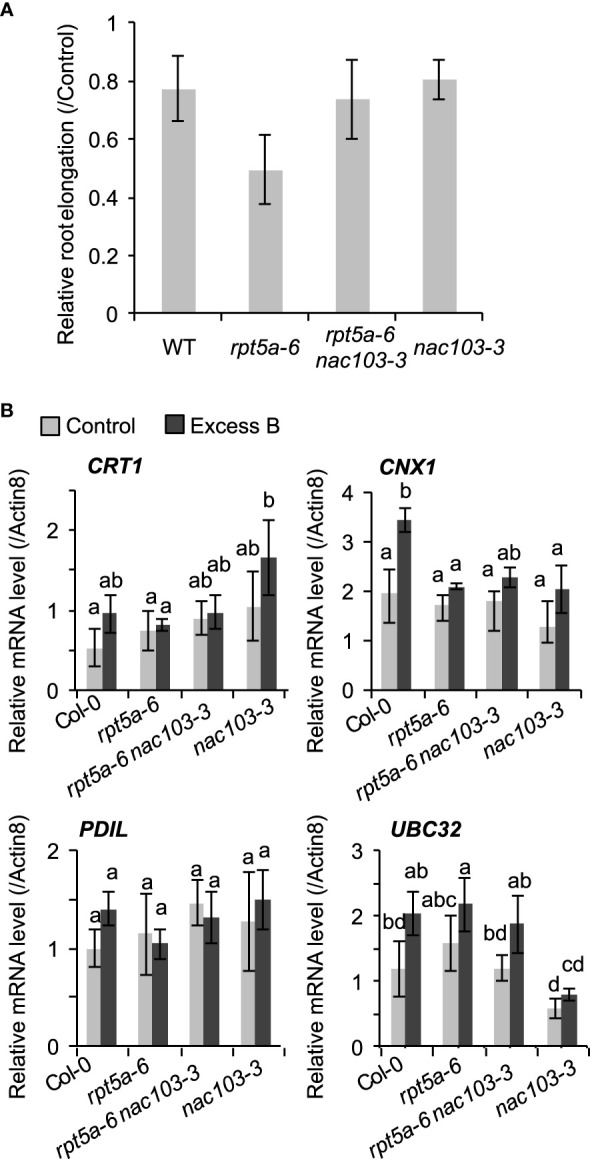
Effect of *nac103-3* mutation on unfolded stress response **(A)** Sensitivity to unfolded protein inducible reagent L-canavanine. Seedlings were precultured in normal conditions (30 µM boric acid) for six days and then transferred to 0.5 µM L-canavanine or control conditions. Root elongation during three days after the transfer was measured and normalized by the average elongation under control conditions. Values represent mean ± standard deviation of measurements of 11-15 seedlings. An asterisk indicates significant difference from the wild type at *p* < 0.05 by Dunnett’s test. **(B)** mRNA accumulation of *NAC103-*downstream unfolded protein response related genes, *CRT1, CNX1, PDIL1* and *UBC32*. Seedlings were grown for 6 days under control conditions (30 µM boric acid) and further 3 days on control or excess boron conditions (3 mM boric acid). Total RNA was extracted from whole root of 10 seedlings and mRNA accumulation was quantified by qRT-PCR. Expression levels were normalized by *Actin8*. Values represent mean ± standard deviation of three biological replicates. Groups sharing the same letters are not significantly different at *p* < 0.05 by Tukey’s test.

The *NAC103* gene has been reported to play a role in the transcriptional regulatory cascade of the unfolded protein response (UPR) (Sun et al., 2013). They have reported that *NAC103* expression is induced by endoplasmic reticulum (ER) stress, which is caused by an accumulation of unfolded proteins *via* direct transcriptional activation by an ER stress signaling regulator bZIP60, and transmits the signal by upregulating the downstream UPR genes. To examine the effects of the *nac103-3* mutation on tolerance to unfolded protein accumulation and root growth, we treated *rpt5a-6* and *rpt5a-6 nac103-3* mutant seedlings with the reagent L-canavanine, which induces protein unfolding *via* its incorporation into the protein; L-canavanine is an analog of arginine and induces protein misfolding (Kurepa et al., 2003). Treatment with 0.5 µM L-canavanine reduced root elongation in the *rpt5a-6* single mutant but not in the *rpt5a-6 nac103-3* double mutant (**Figure 4A**), suggesting that the *nac103-3* mutation suppressed the sensitivity of *rpt5a-6* to the accumulated unfolded proteins.

To assess the involvement of the downstream UPR pathway in excess boron sensitivity with regard to the *rpt5a-6* mutant, we examined the expression levels of the already known NAC103-downstream UPR-related genes: *calnexin* (*CNX*), *calreticulin* (*CRT*), protein disulfide-isomerase 5 (PDI5), and ubiquitin conjugase 32 (UBC32) (Sun et al., 2013). A qRT-PCR analysis detected no significant difference in the expression of the four abovementioned genes and proteins between the *rpt5a-6* and *rpt5a-6 nac103-3* mutants (**Figure 4B**), suggesting that the transcriptional regulation of these genes by *NAC103* is not responsible for excess boron sensitivity of the *rpt5a-6* mutant.

### 3.6 *nac103-3* mutation reduces DNA damage accumulation caused by excess boron stress in the *rpt5a-6* mutant

It has been reported that excess boron stress induces DNA damage in *Arabidopsis* root meristem (Sakamoto et al, 2011), and that the extent of the DNA damage is more severe in the *rpt5a-6* mutant than that in the wild-type plant (Sakamoto et al., 2018). In addition, treatment of *rpt5a-6* with zeocin, a reagent that causes DNA damage, results in a development of a phenotype similar to that of the wild type under excess boron stress conditions (Sakamoto et al., 2018). These results suggest that the susceptibility to DNA damage is a critical factor for excess boron sensitivity in *rpt5a-6.*

**Figure 5:**
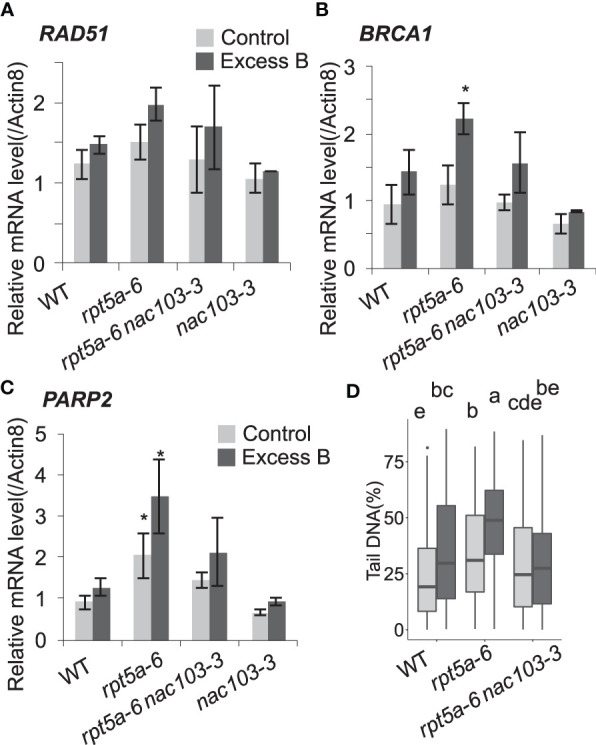
*nac103-3* mutation suppressed DNA damage accumulation in *rpt5a-6* Seedlings were grown for six days under control conditions (30 µM boric acid) and further three days on control or excess boron conditions (3 mM boric acid). **(A–C)** mRNA accumulation of DNA damage maker genes. Total RNA was extracted from whole root of 10 seedlings and mRNA accumulation was quantified by qRT-PCR. Expression levels were normalized by *Actin8*. Values represent mean ± standard deviation of three biological replicates. Asterisks indicate significant difference from the wild type in each condition at *p* < 0.05 by Dunnett’s test. **(D)** Accumulation of DNA damage (double strand break) was quantified with comet assay neutral method. Seedlings were grown for 6 days under control conditions (30 µM boric acid) and further 3 days on control or excess boron conditions (3 mM boric acid). Nuclei were extracted from 1 cm of root tips of approximately 100 seedlings per each condition. The ratios of DNA in tail were calculated by CASP software. For each condition, 125 nuclei were measured. Groups sharing same letters were not significantly different by Steel-Dwass test at *p* < 0.05.

To investigate the role of *NAC103* in this context, we examined the extent of DNA damage in the *rpt5a-6 nac103-3* mutant under the excess boron stress. To estimate the accumulation of DNA damage in roots, the expression levels of genes known to respond to DNA damage, namely *RAD51*, *BRCA1*, and *PARP2*(AT4G02390), were quantified by qRT-PCR analysis (**Figures 5A–C**). Among all genotypes, expression of these genes tended to be higher under excess boron conditions compared to the control condition. Under excess boron conditions, expression of *BRCA1* and *PARP2* was significantly higher in *rpt5a-6* than in wild type, but in *rpt5a-6 nac103-3* it was comparable to wild type. These results suggest that the *nac103-3* mutation suppresses DNA damage accumulation in the *rpt5a-6* mutant. To obtain direct evidence for this, we assessed the extent of DNA damage in root tips using a comet assay (**Figure 5D**). The comet assay revealed that DNA damage accumulation was significantly lower in the *rpt5a-6 nac103-3* mutant than in the *rpt5a-6* mutant, regardless of boron concentration. These results also reveal that the *nac103-3* mutation ameliorated the extent of DNA damage in *rpt5a-6* mutant plants.

### 3.7 *nac103-3* mutation ameliorates DNA damage-induced growth defects in *rpt5a-6* mutant

**Figure 6:**
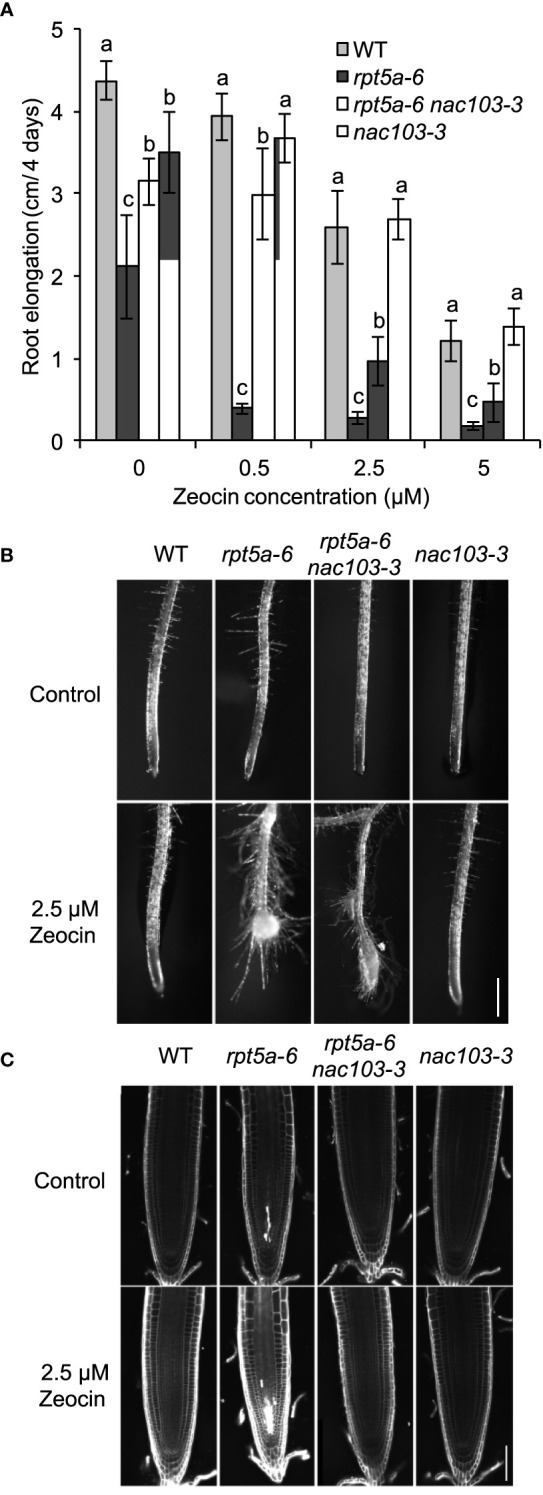
DNA damage sensitivity of *rpt5a-6* is suppressed in *rpt5a-6 nac103-3.* Sensitivity to DNA damage causing regent Zeocin. **(A)** Root elongation under various Zeocin concentrations. Seedlings were precultured the control condition for six days and for further four days with or without Zeocin. Primary root elongation during last four days were measured. Values represent mean ± standard deviation of ten seedlings. For each condition, groups sharing same letters were not significantly different by Tukey-Kramer’s test at *p* < 0.05. **(B)** Primary root tip morphology of 11-day-old seedlings under control or 2.5 µM Zeocin conditions. Bar, 500 µm. **(C)** Confocal microscopy of root meristems. Six-day-old seedlings grown under the control condition were treated with control or 2.5 µM Zeocin conditions for 16 hours. Cell walls and dead cells were visualized with propidium iodide. Bar, 50 µm.

The *rpt5a-6* mutant has been reported to be more susceptible to DNA damage and, more specifically, that caused by radiation or chemical reagents (Sakamoto et al., 2018). To investigate the involvement of *NAC103* in sensitivity to DNA damage, we assessed the root growth of the *rpt5a-6 nac103-3* mutant after treatment with zeocin (**Figure 6**). Treatment with zeocin reduced root elongation in a dose-dependent manner among all tested genotypes (**Figure 6A**). Consistent with previous results, the *rpt5a-6* mutant was more sensitive to zeocin than the wild type, with the former showing a higher root elongation inhibition rate and a more severe morphological alteration, including cell death in root meristems (**Figure 6C**). However, none of these defects were evident in the *rpt5a-6 nac103* double mutant, suggesting that the *NAC103* gene is responsible for the growth defects caused by DNA damage in the *rpt5a-6* mutant.

### 3.8 *nac103-3* mutation alleviates ROS accumulation under excess boron stress in root tips

**Figure 7:**
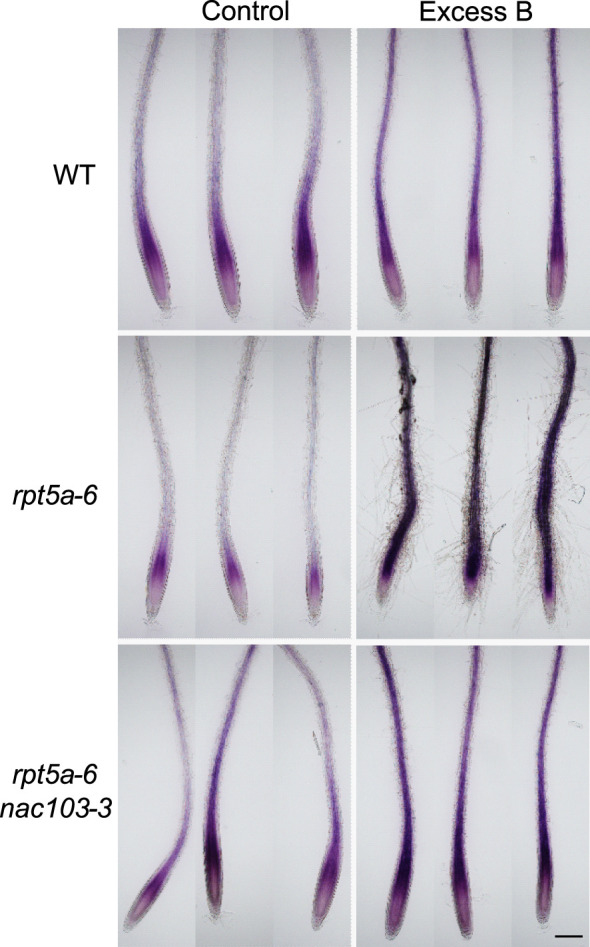
Superoxide accumulation in root tips of *rpt5a-6* and *rpt5a-6 nac103-3.* Seedlings were grown for six days under control conditions (30 µM boric acid) and further three days on control or excess boron conditions (3 mM boric acid). Superoxide was observed as purple stain by nitroblue tetrazolium (NBT) staining. Images from three biological replicates are shown. Bar, 200 µm.

The accumulation of DNA damage motivated us to focus on reactive oxygen species (ROS) because oxidation by ROS is known to be a cause of DNA damage (Amor et al., 1998). To observe ROS accumulation in the mutants, we stained control and excess boron-treated roots with nitroblue tetrazolium (NBT), rendering a purple stain to the superoxide (**Figure 7**). Under excess boron conditions, the stain was more intense in *rpt5a-6* mutant roots than that in the wild-type roots. However, *rpt5a-6 nac103-3* showed lesser staining intensity than both the *rpt5a-6* and the *rpt5a-6 nac103-3* mutants, but it was comparable to that associated with the wild type. These results suggest that NAC103 is responsible for ROS accumulation in *rpt5a-6* under excess boron stress.

### 3.9 *SOG1* is not involved in excess boron sensitivity in *rpt5a* mutants

**Figure 8:**
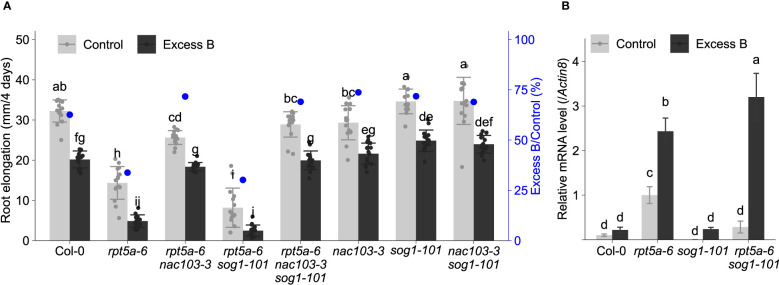
Effect of *sog1* mutation on excess boron sensitivity in root growth. **(A)** Root elongation of rpt5a-6, *nac103-3, sog1-101*, and their double and triple mutants. Seedlings were precultured in normal conditions (30 µM boric acid) for six days and then transferred to excess boron (3 mM boric acid) or control conditions. Root elongation during four days after the transfer was measured. Values are mean ± standard deviation of 15-16 measurements. Groups sharing the same letters are not significantly different at *p* < 0.05 by Tukey-Kramer’s test. **(B)** *NAC103*m RNA accumulation in *sog1-101* mutant. Plants were grown under the same condition as **(A)** and mRNA accumulation was quantified by qRT-PCR. Expression levels were normalized by *Actin8*. Values represent mean ± standard deviation of four biological replicates. Groups sharing the same letters are not significantly different at *p* < 0.05 by Tukey’s test.

As *NAC103* has been suggested to function downstream of *SOG1* transcription factor, a master regulator of DNA damage response genes (Ryu et al., 2019), we examined whether the suppression of excess boron sensitivity by *nac103* mutation is related to SOG1 pathway. Examination of root elongation revealed that excess boron sensitivity of *rpt5a-6 sog1-101* was not significantly different from *rpt5a-6*, suggesting that *sog1-101* mutation does not alleviate excess boron sensitivity of *rpt5a-6* (**Figure 8A**). In addition, excess boron sensitivity of *rpt5a-6 nac103 sog1-101* triple mutant was similar to that of *rpt5a-6 nac103* double mutant. To examine whether induction of *NAC103* by excess boron stress depends on *SOG1*, we examined *NAC103* mRNA abundance in *sog1-101* mutant. Compared to *rpt5a-6* mutant, *rpt5a-6 sog1-101* exhibited even higher *NAC103* mRNA abundance under excess boron conditions, indicating that upregulation of NAC103 under excess B condition does not require *SOG1* (**Figure 8B**). Taken together, these results suggest that contribution of *NAC103* to excess boron sensitivity is likely due to its involvement in pathways other than the SOG1 pathway.

## 4 Discussion

### 4.1 Identification of causal genes as *rpt5a* suppressor mutants

We were able to limit the positional identification of the suppressor mutation in *rpt5a-6 sup1* to the chromosomal region of 353 kb (**Figure 2A**). Whole-genome sequencing revealed DNA sequences in this region; within that region, we found only a single homozygous mutation in the *NAC103* gene. Through independent experiments, we identified 11 mutants exhibiting phenotype similar to that of *rpt5a-6 sup1*. Examination of the *NAC103* sequences revealed that 7 alleles out of the 11 *rpt5a-4* lines carried distinct mutations in *NAC103* (**Figure 2B**). These results suggest that *NAC103* is the gene responsible for the suppression of the *rpt5a* mutant phenotype, but it is fair to consider the possibility that NAC103 is located on so-called hot spot of mutagenesis by EMS. In other projects apart from boron toxicity, we also sequenced other eight mutants, which were also mutagenized in a manner similar to the *rpr5a-6 sup1* mutant. As none of the eight mutants carried a mutation in *NAC103*, the mutations in *NAC103* in *rpt5a* suppressor mutants should not be attributed to a mutagenesis hot spot. In addition, all the *NAC103* mutations in the *rpt5a* suppressor mutants were located within the N-terminus half of the gene, which was annotated as the NAC domain, a conserved DNA binding domain (**Figure 2**). Thus, we suggest that the mutations can be attributed to the functionality of the proteins rather than chromosomal location. In the complementation tests, the introduction of the *proNAC103*-*NAC103*-*GFP* construct partially reversed the phenotype of the *rpt5a-6 sup1* mutant to that of the *rpt5a-6*. The incomplete recovery could be attributed to possible effects GFP-tag fused to C-terminus of NAC103, rather than low expression levels of the transgene, because *NAC103* mRNA levels in the transgenic lines were comparable or higher than in *rpt5a-6* (**Supplementary Figure 4**). However, given that the changes in phenotype were observed, the transgene is at least partially functional, and *NAC103* gene is likely to be involved in the root growth defects in the *rpt5a-6* mutant under boron stress. Taken together, we conclude that the mutation in NAC103 is responsible for the suppression of the excess boron-sensitive phenotype of *rpt5a* mutant.

### 4.2 Involvement of *NAC103* in excess boron sensitivity

The NAC family is one of the largest families of plant-specific transcription factors (Ooka et al., 2003). *NAC103* has been characterized in the context of its expression in response to unfolded proteins levels (Sun et al., 2013). They showed that the NAC103 protein is a substrate of the 26S proteasome, and that it is maintained at a low concentration under normal conditions; furthermore, it is stabilized under unfolded protein stress.

The upregulation of NAC103 in turn activates the transcription of downstream unfolded protein response genes such as molecular chaperones [namely, *calnexin* (*CNX*), *calreticulin* (*CRT*), and *protein disulfide-isomerase 5* (*PDI5*), and *ubiquitin conjugase 32* (*UBC32*)]. In our study, mRNA accumulation of those *NAC103* downstream genes was not significantly different between the *rpt5a-6* and *rpt5a-6 nac103-3* mutants (**Figure 4B**), suggesting that these genes were not responsible for the suppression of the *rpt5a-6* phenotype in *rpt5a-6 nac103-3* mutant plants. In addition, our previous study demonstrated that excess boron treatment does not affect accumulation of poly-ubiquitinated proteins, which include unfolded proteins to be degraded by proteasome (Sakamoto et al., 2018) Thus we can deduce that excess boron treatment does not induce unfolded protein significantly. These together suggest that it is not unfolded protein response pathway that is critical for excess boron response.

It has been reported that NAC103 interacts with VND, a master regulator of xylem vessel differentiation that functions as a transcription activator (Yamaguchi et al., 2015). According to their yeast two-hybrid assay, NAC103 can form hetero-complexes with various other NAC transcription factors, such as VND1, VND2, VND3, VND7, NAC1, CUC2, and VNI1. This suggests that *NAC103* can play multiple roles in different cellular processes through its interaction with different counterparts. They also indicated that *NAC103* may regulate cellular stress response by binding with other NAC domain proteins. This point of view provides possible explanation for our observation that overexpressing *NAC103* alone did not affect root growth under excess B conditions. If NAC103 requires counterpart(s) to be functional under excess B conditions, overexpressing NAC103 could affect the phenotype only when enough number of counterpart proteins are available. Identification of the potential counterpart in future study would clarify this point.

Assuming that the mutations in NAC103 are loss of function, our results suggest that accumulation of NAC103 has negative effects on root growth, because *nac103* mutations suppressed the growth defects of *rpt5a* mutants. Considering that *NAC103* is a substrate of the 26S proteasome and that *RPT5A* is suggested its involvement in recognition of poly-ubiquitinated proteins in the Ub–proteasome pathway (Lam et al., 2002), it can be speculated that control of NAC103 protein accumulation was altered in *rpt5a* mutants. This should be confirmed by observation of NAC103-GFP in *rpt5a-6 nac103-3*. However, under our experimental conditions, we could not detect GFP fluorescence in transgenic plants introduced with the *proNAC103-NAC103-GFP* construct, even after treatment with MG132, which stabilizes the NAC103 protein (Sun et al., 2013). Could be because we did not have enough expression to observe fluorescence as the construct was driven by *NAC103* own promoter, not by a strong constitutive promoter.

### 4.3 Possible mechanisms of excess boron sensitivity in roots

In *Brassica napus*, homologue of *NAC103*, *BnaNAC103* was identified with 75.4% identity and 76.5% similarity at the nucleotide level (Niu et al., 2014). Furthermore, they revealed that in *Nicotiana benthamiana* leaves, overexpression of *BnaNAC103* causes ROS accumulation and induces cell death. They mentioned in the report that whether *NAC103 in A. thaliana* (*AtNAC103*) regulates cell death is unknown yet. However, it is likely that *AtNAC103* has a similar negative function considering that ROS accumulation and cell death in the *rpt5a-6* mutant under excess boron stress was suppressed in *rpt5a-6 nac103-3* (**Figure 7**). Although how *NAC103* could induce ROS remains unclear, the simplest hypothesis should be that *NAC103* may upregulate ROS producing enzyme, or negative regulator of ROS scavenger, considering that NAC103 is a transcription factor.

The observed ROS accumulation and cell death under excess boron conditions can be interpreted in two ways: ROS induces cell death, or, cell death releases ROS from cells. Although further careful investigation is needed to dissect this cause-effect relationship, our results allow us to infer it. After three days of excess boron treatment, strong NBT staining was observed throughout most of the root area in *rpt5a* mutant, suggesting that ROS is accumulated in whole root (**Figure 7**). On the other hand, under the same treatment condition, cell death was observed only in stele cells in the meristem (**Supplementary Figure 1**). This suggests that, at least in most of the root tissue, ROS accumulation does not require preceding cell death. Thus, we deduce that it is more likely that ROS is the cause of cell death, rather than ROS accumulation is the result of cell death.

Several independent microarray analyses have revealed that *NAC103* expression is induced during conditions of DNA damage caused by γ-ray exposure or treatment with hydroxyurea or bleomycin (Culligan et al., 2006; Yoshiyama et al., 2009; Cools et al., 2011; Yi et al., 2014). Ryu et al. (2019) reported that *NAC103* is induced by SOG1, a NAC transcription factor known to regulate DNA damage response gene, and is partially responsible for DNA damage response phenotypes. They revealed that *NAC103* interacts with the putative promoter regions of *Rad51, PARP2* (AT4G02390, which is referred to as *PARP1* in Ryu et al., 2018)*, RPA1E, BRCA1* and *At4g22960*, suggesting that *NAC103* is a putative *SOG1*-dependent DNA damage response regulator. However, our results indicate that *NAC103* has some functions in *SOG1*-independent pathway, because *NAC103* induction under excess boron condition was *SOG1*-independent and *sog1* mutation did not suppress excess boron sensitivity of *rpt5a* mutant (**Figure 8**). In our study, the *nac103-3* mutation suppressed both DNA damage and ROS accumulation in the root tips of *rpt5a-6* mutant plants in the presence of excess boron. This indicates that irregular accumulation of *NAC103* in the proteasome mutant *rpt5a-6* induces DNA damage and ROS accumulation under excess boron stress, suggesting the role of *NAC103* in DNA damage induction or suppression of DNA repair and/or ROS production or radical scavenging activity.

**Figure 9:**
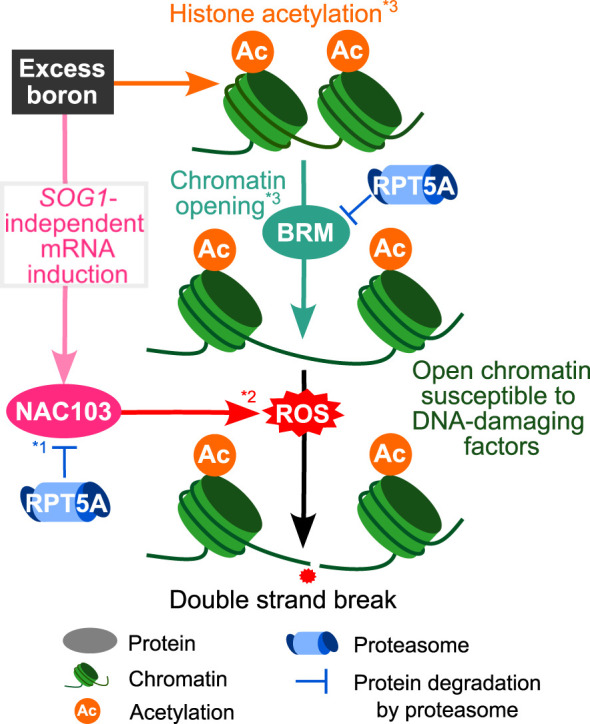
Suggested mechanisms for DNA damage caused by excess boron stress in wild type plants. Excess boron stress induces *NAC103* mRNA in *SOG1*-independent manner. As NAC103 protein is degraded *via* proteasome pathway (*^1^ Sun et al., 2013), mutation in proteasome (RPT5A) should result in over-accumulation of NAC103 protein. Over-accumulation of *NAC103* is likely to induce ROS accumulation (*^2^ Niu et al., 2014). ROS is a major cause of DNA damage. Together with the known BRAHMA(BRM)-dependent pathway in which excess boron stress induces histone acetylation to loosen chromatin, making DNA susceptible to DNA-damaging factors (*^3^ Sakamoto et al., 2018), the NAC103 dependent pathway plays a crucial role in growth defects under excess boron conditions.

In summary, our study sheds light on the mechanisms underlying excess boron-induced DNA damage accumulation and growth reduction (**Figure 9**). Our previous study revealed that excess boron causes histone acetylation and BRM-dependent chromatin opening (Sakamoto et al., 2018). This is thought to increase the susceptibility to DNA damaging factors. On the other hand, our study suggested that NAC103 is involved in a downstream pathway, which is likely responsible for the accumulation of a DNA damaging factor, ROS. Excess boron stress induces NAC103 mRNA in *SOG1*-independent manner. In the wild type, NAC103 protein level is likely maintained at low level by the 26S proteasome, which would ameliorate excessive ROS accumulation. However, an alteration in the 26S proteasome of the *rpt5a-6* mutant can lose this maintenance, which may in turn result in accelerated ROS accumulation. This would result in increased DNA damage, which could be the direct cause for root growth inhibition. *rpt5a-6* mutation also induced *NAC103* mRNA, but considering that the induction was observed even under control conditions, this mRNA induction itself is likely due to a boron-independent mechanism.

## Data availability statement

The original contributions presented in the study are included in the article/**Supplementary Material**. Further inquiries can be directed to the corresponding authors.

## Author contributions

NS: Conceptualization, investigation, resources, writing – original draft, visualization, funding acquisition. TS: Conceptualization, investigation, resources, writing – review and editing, visualization, funding acquisition. TK: Investigation, resources. RT: Investigation, writing – review and editing. KY: Formal analysis. SSh: Formal analysis. MY: Investigation. MH: Investigation, resources. SSa: Investigation, resources, funding acquisition. TF: Conceptualization, resources, writing – review and editing, supervision, funding acquisition. All authors contributed to the article and approved the submitted version.
