# Supplementary material for: NAC103 mutation alleviates DNA damage in an Arabidopsis thaliana mutant sensitive to excess boron
Source: Front Plant Sci. 2023 Mar 29;14:1099816. doi: 10.3389/fpls.2023.1099816 (PMC10090426; doi:10.3389/fpls.2023.1099816)
Supplement: Supplementary file 1 [file DataSheet_1.pdf]

Supplementary Material

1 Supplementary Figures and Tables

1.1 Supplementary Figures

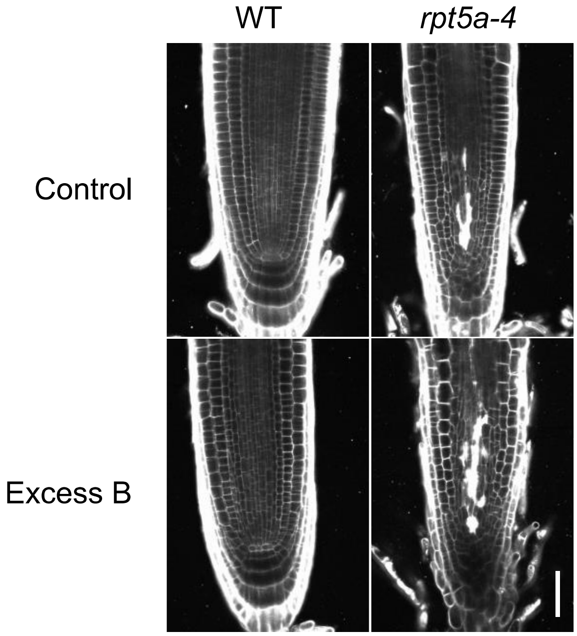

Supplementary Figure 1. Cell death observed in *rpt5a* mutant under excess boron conditions. Confocal microscopy of root meristems. Six-day-old seedlings grown under the control condition were treated with control or excess boron conditions for three days. Cell walls and dead cells were visualized with propidium iodide. Bar, 50 µm.

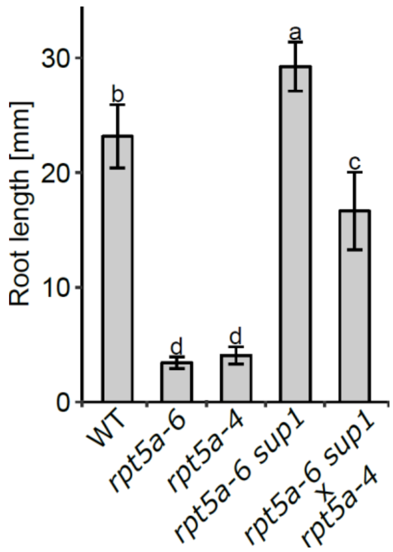

Supplementary Figure 2. Primary root length of *rpt5a-6 sup1* x *rpt5a-4 F1* under excess boron conditions. Seedling were grown for 10 days under excess boron (3 mM boric acid) condition. Values are mean ± standard deviation of 4-5 biological replicates. Groups sharing the same alphabets are not significantly different at $p < 0.05$ by Tukey-Kramer's test.

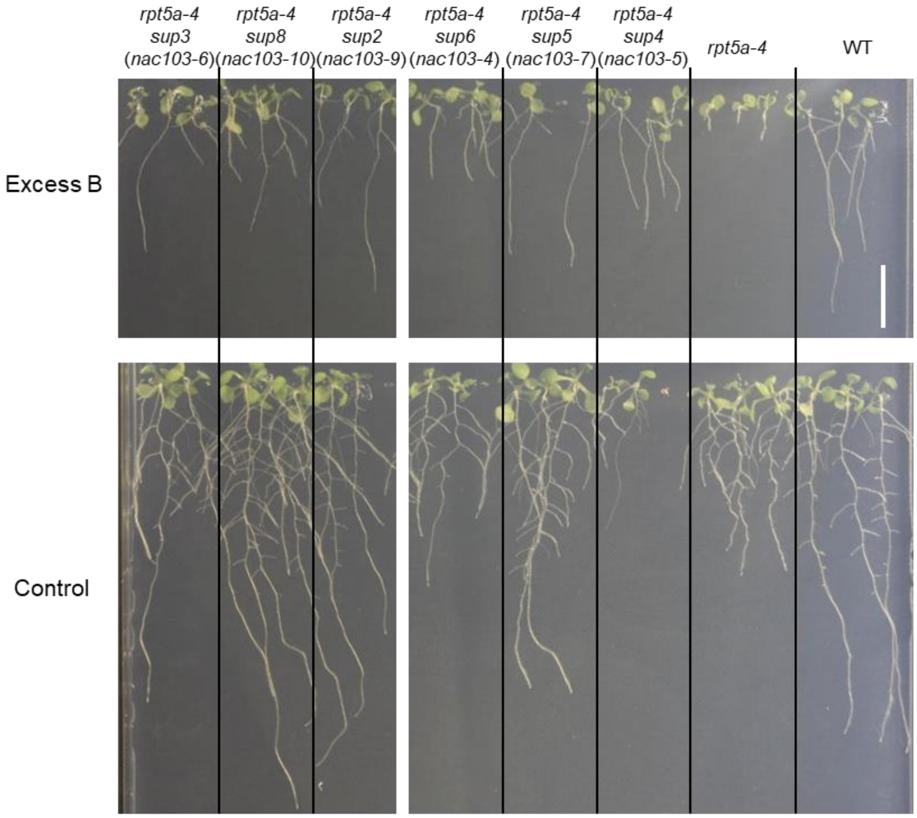

Supplementary Figure 3. Phenotype of *rpt5a-4* suppressor mutants. *rpt5a-4* suppressor mutants were grown for 10 days under excess boron (3 mM boric acid) or control (30 µM boric acid) conditions. Scale bar, 10 mm.

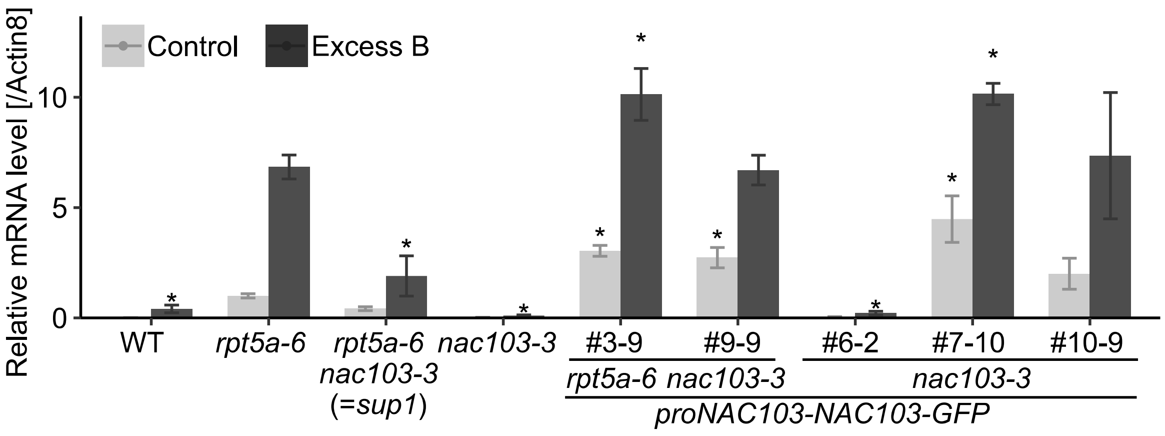

Supplementary Figure 4. *NAC103* mRNA accumulation in *rpt5a-6* mutant and *NAC103* overexpression lines. Seedlings were precultured in normal conditions (30 µM boric acid) for eight days and then grown on excess boron (3 mM boric acid) or control conditions for five days. Total RNA was extracted from whole root and *NAC103* mRNA accumulation was quantified by qRT-PCR. Expression levels were normalized by *Actin8*, and values are expressed relative to *rpt5a-6* under control condition. Values represent mean ± standard deviation of three biological replicates. Asterisk indicate significant difference from the *rpt5a-6* in the same condition at $p < 0.05$ by Dunnett’s test.

1.2 Supplementary Tables

Supplementary Table 1.

SNP marker	rpt5a-6_BfuAI_F	CAGGAGAAGATTAAACTCAAC	
	rpt5a-6_R	CATGTAATTATTGTTGCCAG	
	nac103-1_F	AAAGAAGTATCCCAAAGGCG	*1
	nac103-1_R	ATCGTCATTACCTGAGGAAC	
Construction	NAC103_CDS_F	CACCATGGGGAAAACTAACTTG	
	NAC103_CDS_R	TTAATCGTCCTTAGTCTGAC	
	NAC103ATG-2k_F	CACCCACTCTTCACACATATAC	
	NAC103CDS-TAA_R	ATCGTCCTTAGTCTGACCGTTG	
	NAC103upperATG_R	TGGAAGACAAGGGGAAAAC	*1
	NAC103_ATG-200_F	GATATCTGCAAGAAGAATCC	*1
	NAC103_ATG-1219_F	CTTTCTCAAACCTTGACAAC	*1
	NAC103_ATG-652_R	AGTGAGTGATGGGGTTTAGG	*1
Col-0/Ler marker	MTG13_F	ATTTGCAAGTGTCGATGGAA	
	MTG13_R	TGGTTTCAAGCCTCTGATAATTT	
	MYN8_F	GTCATGCTCATGAACCTGTTTT	
	MYN8_R	TTCACAAGGGATTAAGAACCAA	
	MLE2_F	AAACTTTTGCCCCTTCCTG	
	MLE2_R	TGATGATTHHHTTGATTCAAA	
	MGI19_F	TGGTCACACTCAAACAAGCA	
	MGI19_R	CACCATCACCACCAGTATGC	
	T12B11_F	TATCGGTGATGGTGGTGTTG	
	T12B11_R	CACAGTGTCCCCAAAGTCA	
qRT-PCR	NAC103RTPCR_F	CCTCAAGTATCTCTCGATGG	
	NAC103RTPCR_R	GATGTCTTACCTCATTATGG	
	Actin8_RT_F	GCCAGATCTTCATCGTCGTG	
	Actin8_RT_R	TCTCCAGCGAATCCAACCTT	
	BRCA1_RT_F	CCATGTATTTTGCAATGCGTG	
	BRCA1_RT_R	TGTGGAGCACCTCGAATCTCT	
	RAD51_RT_F	CGAGGAAGGATCTCTTGCAG	
	RAD51_RT_R	GCACTAGTGAACCCCAGAGG	
	PARP1_RT_F	GCTTTGGGAGACATGAATGAAC	*3 *4
	PARP1_RT_R	AAGTGGAACAACAACACCGTCT	*3 *4
	CRT1_RT_F	AGACCTTAGTCTTCCAATTCTC	*2
	CRT1_RT_R	CCATTGTAAGTAAGGATAGCATG	*2
	CNX1_RT_F	ATGAGACAACGGCAACTATTTTCC	*2
	CNX1_RT_R	CCATAATCCTCATGTCCTTCACT	*2
	PDIL_RT_F	CTCGTGAAGCTGAGGGTATTG	*2
	PDIL_RT_R	TGTGCGAAATCTAACTCAGAG	*2

UBC32_RT_F	CAGACACCATAGTTGATGAT	*2
UBC32_RT_R	ACCATGATAGCGATTGTGAG	*2

*1 Used for sequencing analysis for vector validation.

*2 Sun et al., 2013

*3 Ricaud et al., 2007

*4 For *PARP2.* In this report, *PARP2 corresponds to* AT4G02390, which is referred to as PARP1 in Ricaud et al., 2007
